# Sugar, fat, and protein: new insights into what T cells crave

**DOI:** 10.1016/j.coi.2015.01.015

**Published:** 2015-02-06

**Authors:** Greg M. Delgoffe, Jonathan D. Powell

**Affiliations:** 1 Tumor Microenvironment Center, Department of Immunology, University of Pittsburgh Cancer Institute, Pittsburgh, PA; 2 Sidney-Kimmel Comprehensive Cancer Center, Department of Oncology, Johns Hopkins University School of Medicine, Baltimore, MD

## Abstract

T cell activation and differentiation is a complex process that has evolved beyond the two-signal model to a number of varied and opposing inputs that must be interpreted to make a cell fate decision. While stimulation through the TCR, costimulatory, and cytokine receptors is required, metabolic signaling has emerged not only an activation signal, but one that can influence and shape differentiation. Recent findings have revealed unappreciated roles for glucose, fatty acids, and salt in the function of many T cell subsets. In this review, we will highlight the latest advances in the burgeoning field of immunometabolism, focusing on how the menu of T cell fuels has expanded.

## Introduction

During his studies of tumor cells, Otto von Warburg hypothesized that a key transformation event in the development of cancer was the ability to shift their metabolism to support their enhanced proliferation[1]. He observed that, even though tumor cells are in a relatively oxygen-rich environment, they preferentially ferment glucose, producing lactate, rather than consume oxygen and undergo respiration. This type of metabolism, aerobic glycolysis or the “Warburg effect”, is a key characteristic of many cancers[2].

During an immune response, T cells can expand 10–100000 fold during their initial expansion, and need fuel and metabolic intermediates to support their proliferation. Thus, upon activation, conventional T cells, participate in aerobic glycolysis, just like cancer cells[3]. While complete glycolysis, fermenting glucose into lactic acid, is a relatively inefficient means of producing ATP, it is considered to be favorable to highly proliferative cells, as it frees up intermediates for building new cellular components (membranes, proteins, and nucleotides), favoring cell division[4]. In stark contrast, naïve and memory T cells must be able to survive for years, in order to support primary and secondary responses, without undergoing any substantial proliferation. During these periods of quiescence, T cells have been shown to primarily use mitochondrial metabolism to support their survival, utilizing fatty acids, amino acids, and glucose to generate ATP through the TCA cycle and oxidative phosphorylation[5].

Thus, T cells must be able to modulate their metabolism in order to switch between these two distinct proliferative modalities. However, the metabolic requirements of T cells are extraordinarily complex and can vary heavily between individual populations and subsets. Recent studies have attempted to dissect the interplay between fuel and function. In this review, we will focus on these recent insights into what kind of fuel T cells use and how nutrients and nutrient sensors can shape the immune response.

## Sugar: memory cells may lack a sweet tooth

Glucose is the predominant (and most studied) fuel source used by somatic cells to generate ATP. Initial studies in cancer cell and T cell metabolism focused on the metabolic “switch” of Warburg metabolism: what factors, signaling pathways, or transcriptional programs could induce this shift away from oxidative metabolism. However, it is becoming increasingly clear that the bioenergetic fate of glucose is not the only factor in cellular metabolism, and that Warburg metabolism may not be the ideal mechanism for generating strong, durable immunity.[6] While T cells dynamically regulate these pathways, and upregulate both oxidative phosphorylation and glycolysis during activation, it is the ability for the cells to engage glycolysis that is critical for the translation and secretion of some cytokines, especially interferon gamma (*Ifng*).[7]

While the incredible proliferation of an expanding T cell is required to generate an army of effector cells, another important goal of that initial activation is the development of potent memory. Like most highly proliferative cells, Warburg metabolism spares cellular biomass from catabolic metabolism to promote T cell expansion. However, it has not been entirely clear how glycolytic programs might help or hinder T cells’ transition into memory phase. A recent study suggests that the shift to glycolytic metabolism is associated with terminal differentiation and less memory progenitor cells during initial antigen encounter, and that enforced glycolysis can inhibit the memory response [8]. Further, inhibition of glycolysis using 2-deoxyglucose during activation can augment memory cell generation, suggesting that aerobic glycolysis is not necessarily a preferred metabolic modality for T cells. Indeed, inhibition of glycolysis can inhibit the activation of mTOR, a critical nutrient sensor important in dictating effector versus memory fates[9]. Supporting the idea that glycolytic machinery might promote effector T cell differentiation, recent data suggest that glyceraldehyde-3-phosphate dehydrogenase (GADPH), an enzyme that has critical activity in glycolysis, also has functions as an RNA-binding protein for *Ifng*[7]. This study showed that in T cells, GAPDH could bind to the AU-rich elements of the 3′UTR of *Ifng* mRNA, repressing transcription, revealing a novel way that metabolism can directly modulate transcription in T cells[7]. At some point during expansion, a cell needs to shift back into an oxidative metabolism in order to preserve and carry out cellular functions outside of proliferation. Reliance on a single source of fuel may lead to skewed or abnormal immune response; invariably, catabolic metabolism will be required to generate intermediates, second messengers, and high ATP levels in order to carry out diverse cellular functions.

Thus, the control and timing of glucose uptake could have major impact on the generation of memory during T cell expansion. The glucose transporter *Glut1* is dynamically regulated and critical for glucose influx into T cells, at the levels of transcription, post-translational modification, and cellular localization[10, 11]. Indeed, recent studies have shown that, not only is it the dominant glucose transporter in CD4^+^ conventional T cells, but that it seems to be dispensable for T_reg_ cell function, consistent with previous data suggesting T_reg_ cells do not rely on glycolytic programs[12]. This could potentially be due to the fact that T_reg_ cells, especially those that are highly suppressive, seek to restrain Akt activation in order to maintain their stability and suppressive function[13]. As Akt is important for *Glut1* activity and trafficking, this may explain why T_reg_ cells have limited glycolysis[11, 14]. However, while such metabolic programs may promote the generation and maintenance of T regs, data suggests that “effector-like” T regs may indeed employ increased glycolytic programs similar to their T conventional effector counterparts[15–17].

In addition, Akt may not be the only way T cells control glucose uptake. Other studies have expanded on T cell regulation of glucose transport, including GCN2, a target of the indoleamine 2,3-dioxygenase (IDO) pathway, and leptin, an adipokine that plays a central role in systemic metabolism[18, 19]. These two signaling pathways can modulate *Glut1* and *Glut3* expression in an Akt-independent manner, suggesting that many distinct pathways may be able to modulate the glycolytic machinery. Future studies should seek to determine how and when, during the effector phase of the immune response, T cells limit glucose uptake to prevent unrestrained glycolysis. Some of these effects may be mediated through the adenosine monophosphate activated protein kinase (AMPK). AMPK acts a critical sensor of energy charge; when AMP is high in the cell, AMPK can be activated through LKB1. AMPK suppresses glycolytic and lypolytic programs, and inhibits mTOR activation directly or indirectly through tuberous sclerosis complex (TSC)[20, 21]. Thus, it stands to reason that AMPK would be important for suppressing unrestrained glycolysis and thus promoting T cell memory. Indeed, using a floxed allele of *Ampka1*, AMPK has been shown to be a critical regulator of T cell memory [22]. Cells deficient in AMPK had a robust effector response, but failed to transition to memory. While the mechanism is still unclear, these studies suggest that, at some crucial timepoint, glucose uptake and glycolysis *must* be attenuated in order to effectively transition to a long-lived memory phenotype.

## Fat: fuel for the slow burn

Lipids represent a bioenergentically rich fuel source for cells; the oxidation of long fatty acid chains generates acetyl-CoA for the TCA cycle, resulting in the generation of large amounts of ATP[23]. However, in heavily proliferative cells, the utilization of this fuel would come at a cost: fats are vital for the generation of cell membranes and second messengers. So, the utilization of lipids could serve as an important fuel source during periods of long-term T cell quiescence, as needed for naïve and memory cells, but would need to be suppressed during T cell expansion[6]. Fatty acids enter cells a number of ways to undergo oxidation. This pathway consists of a transporter, Octn2 (*Slc22a5*), to take up free fatty acids (FFA), coupled to an activation step that couples FFAs to coenzyme A to form an acyl-CoA, a carnitine shuttle which translocates the fatty acids into the mitochondria through the rate-limiting enzyme carnitine palmitotransferase I (*Cpt1a*), and the breakdown into acetyl-CoA molecules to feed into the TCA cycle inside the mitochondria[23].

Previous studies have suggested that memory CD8^+^ T cells utilize enhanced fatty acid oxidation to function[24]. It was assumed that memory cells took up FFA from their environment, generating a large amount of ATP to fuel their basal functions during quiescent states. However, this had not been proved formally and the source of cellular fatty acids remains unclear[25]. Intriguingly, recent work suggests that memory T cells utilize cell-intrinsic fatty acids they themselves synthesized from carbohydrate sources[26]. In other words, cells utilize glucose to generate fatty acids then feed those fatty acids into the beta oxidation cycle to generate ATP. This “futile cycle” seems paradoxical; cells must utilize ATP and reducing equivalents in order to generate fatty acids that they will then break down for ATP. It has been hypothesized that the futile cycle keeps metabolic machinery “primed” during long periods of quiescence, preventing loss of mitochondria. It will be important to determine how exactly this futile cycle is initiated, what purpose it might serve, and how it might be broken? For instance, other studies have shown that some types of CD8^+^ T cells, especially alloreactive T cells in GVHD, readily take up and oxidize exogenous free fatty acids[27]. Thus, a question remains as to whether this futile cycle metabolism is important for memory cells specifically or is a more common phenomenon in T cell biology. Along these lines, the cellular control and flux of fatty acids has also been shown to be important for CD4^+^ helper T cells, specifically the balance between suppressive T_reg_ and IL-17-producing T_H_17 cells[28]. Acetyl-CoA carboxylase 1 (ACC1), a key regulator of fatty acid synthesis, has been shown to both promote T_H_17 cell generation and inhibit T_reg_ cell generation[29]. T_H_17 cells seem to generate their own fatty acids (reminiscent of a futile cycle), while T_reg_ cells take them up from their surrounding environment. However, it is unclear from this study whether fatty acids are being oxidized for ATP or simply being utilized as biomass. Thus, the importance of these futile cycles for fatty acid flux in helper T cells remains to be full understood. Importantly, these findings also suggest that other futile cycles may exist for other metabolites.

## Choosing from the menu: metabolites dictate T cell fate through nutrient sensing

As T cells begin to undergo activation and differentiation, they require metabolically rich conditions in order to support their proliferation. Thus, it stands to reason that the ability to *sense* these conditions could provide a crucial survival or differentiation signal. The macrolide rapamycin, while a poor antibiotic, was shown in the 1990s to be a potent immunosuppressant, whose target protein, mTOR, was revealed to be an evolutionarily conserved protein kinase[30, 31]. mTOR was subsequently shown to play a major role in the ability of somatic cells to sense nutrients, including glucose, amino acids, energy charge, and growth factors, and to use these inputs to make cell fate decisions[32]. In T cells, blockade of mTOR with rapamycin, metabolic deprivation, or genetic deletion, results in a tolerogenic state, characterized by T cell anergy[33–35] and regulatory T cell differentiation[36–39]. In addition, it is now appreciated that, for CD8^+^ T cells, mTOR activation delivers a signal that promotes effector cell generation and function[9]. Alternatively, when mTOR is inhibited with low doses of rapamycin during T cell activation in vivo, T cell memory is dramatically improved, both in quality and quantity[9, 40]

Research in recent years has dissected further the role of mTOR signaling in regulating T cell differentiation and function[41, 42]. Signaling via mTOR can proceed via two protein complexes, mTOR complex 1 (mTORC1) and mTOR complex 2 (mTORC2), which are characterized by distinct scaffolding proteins and downstream substrates[43]. Our group and others have demonstrated a selective role for Rheb-mediated mTORC1 activation in promoting Th1 and Th17 differentiation and a role for mTORC2 in promoting Th2 differentiation[44]. Alternatively, the absence of mTOR promotes T_reg_ cell differentiation even under conditions that would normally lead to T cell activation[36]. Interestingly, recent studies using raptor (a critical scaffolding protein of mTORC1)-deficient T cells suggest a role for raptor-mediated mTORC1 activity in controlling all T helper cell differentiation and function[17, 45]. Indeed, raptor-deficient T cells demonstrate defects in Th1, Th2, Th17 and T_reg_ cell function. The floxed allele of *Rptor* used in each of these studies results in a more profound loss of mTORC1 activity, unlike previous studies utilizing rapamycin or deletion of *Rheb*. This may indicate that distinct functions of mTORC1 exist. *Some* mTORC1 activity is necessary for generic cellular functions (translation initiation, escape from quiescence, and some metabolic programming), but that heightened mTORC1 is required for activating selective differentiation programs. Deconvoluting these two roles for mTORC1 will be critical to fully understanding how this critical nutrient sensor controls T cell fate.

To this end, a next critical step toward dissecting the role of mTOR in T cell differentiation and function is to determine the specific mTORC1 and mTORC2 substrates that promote this regulation. One downstream substrate of mTORC2, the serum and glucocorticoid regulated kinase (SGK1)[46, 47] has been shown to play a major role in promoting Th2 differentiation[48]. T cells deficient in SGK1 demonstrate defective Th2 differentiation. Furthermore, such cells tend to secrete Th1 cytokines even when skewed under Th2 conditions. Interestingly, SGK1 has also been implicated in promoting the generation of pathogenic Th17 T cell differentiation through its regulation of the IL-23 receptor[49].. These studies and another further implicated SGK1 in promoting the ability of T cell to respond to salt concentrations.

## Other nutrient sensors cross paths with mTOR

Additional work has focused on how biochemical pathways downstream of mTOR sensing intersect with programs that regulate metabolism and T cell function[41]. The transcription factor Myc is activated in response to TCR stimulation and has a wide variety of cellular functions. Myc has been revealed as a critical regulator upstream of glycolysis and glutaminolysis[50]. In its absence, T cells fail to engage glycolysis or glutamine metabolism, proliferate, or acquire an effector phenotype. Glutaminolysis in T cells is required for the synthesis of polyamines, which are important for a wide variety of cellular functions. Furthermore, the orphan receptor estrogen-related receptor alpha (ERRα) also appears to be a sensor and transcriptional regulator required for effector T cell transitions[51]. In its absence, effector T cells cannot proliferate or modulate their metabolic programming, but T_reg_ cells do not require ERRα to function.

Another sensor that has found a new appreciation in T cells is hypoxia-inducible factor 1, or HIF1α. As oxygen is essential for oxidative phosphorylation and other cellular functions, it is not surprising that a sensor for hypoxia would be important in cellular function. Recently, research from several groups have revealed a complex role for HIF1α in T cell differentiation and function, both along the T_reg_/T_H_17 cell axis as well as in the differentiation and migration of CD8^+^ T cells[52–56]. Interestingly, in spite of its name, HIF1a appears to play a critical role in regulating T cell activation and differentiation independent of its ability to sense oxygen. Nonetheless, these studies suggest that a T cell’s ability to sense an oxygen rich environment may also be critical in making cell fate decisions, and that hypoxic environments (such as tumors) may promote the tolerogenic differentiation of T_reg_ cells.

Finally, new energy sources have been revealed in T cell biology. While autophagy has been previously thought to be a process associated with quality control, cellular aging, or even a prelude to cell death, it has become clear recently that autophagy is a vital cellular process that is not only required for some cell functions, but also can be a source of fuel[57]. By undertaking a genetic approach using T cell-specific deletions of *Atg7* or *Atg5*, key genetic regulators of autophagy, two different groups found that, while autophagy is dispensable for effector T cell proliferation and function, T cells lacking autophagy could not transition into the memory pool and subsequently died by apoptosis[58, 59]. Notably, many forms of autophagy are induced upon mTOR inhibition, which has been shown to promote the effector-to-memory T cell transition. Future studies will need to address what exactly occurs during T cell autophagy, and whether or not autophagy is, indeed, a source of fuel for T cells or has some sort of other function in T cell biology.

In summary, metabolism represents critical cellular processes that can have wide-reaching effects on T cell biology, not simply a switch thrown to decide the fate of glucose. As our appreciation of the role of fuel sources for T cell differentiation and function grows, so too must our appreciation of how distinct microenvironments and tissues might modulate nutrient availability. Nutrient sensing is comprised of ancient signaling pathways heavily conserved from yeast to man. These conserved pathways are emerging as the key components of cellular programs which coordinate T cell differentiation and function and metabolic needs. Understanding and dissecting the evolution of these pathways will not only reveal fundamental insight about T cell biology, but likely identify metabolic targets that could be used to modulate the immune response in autoimmunity or cancer.

## Figures and Tables

**Figure 1:**
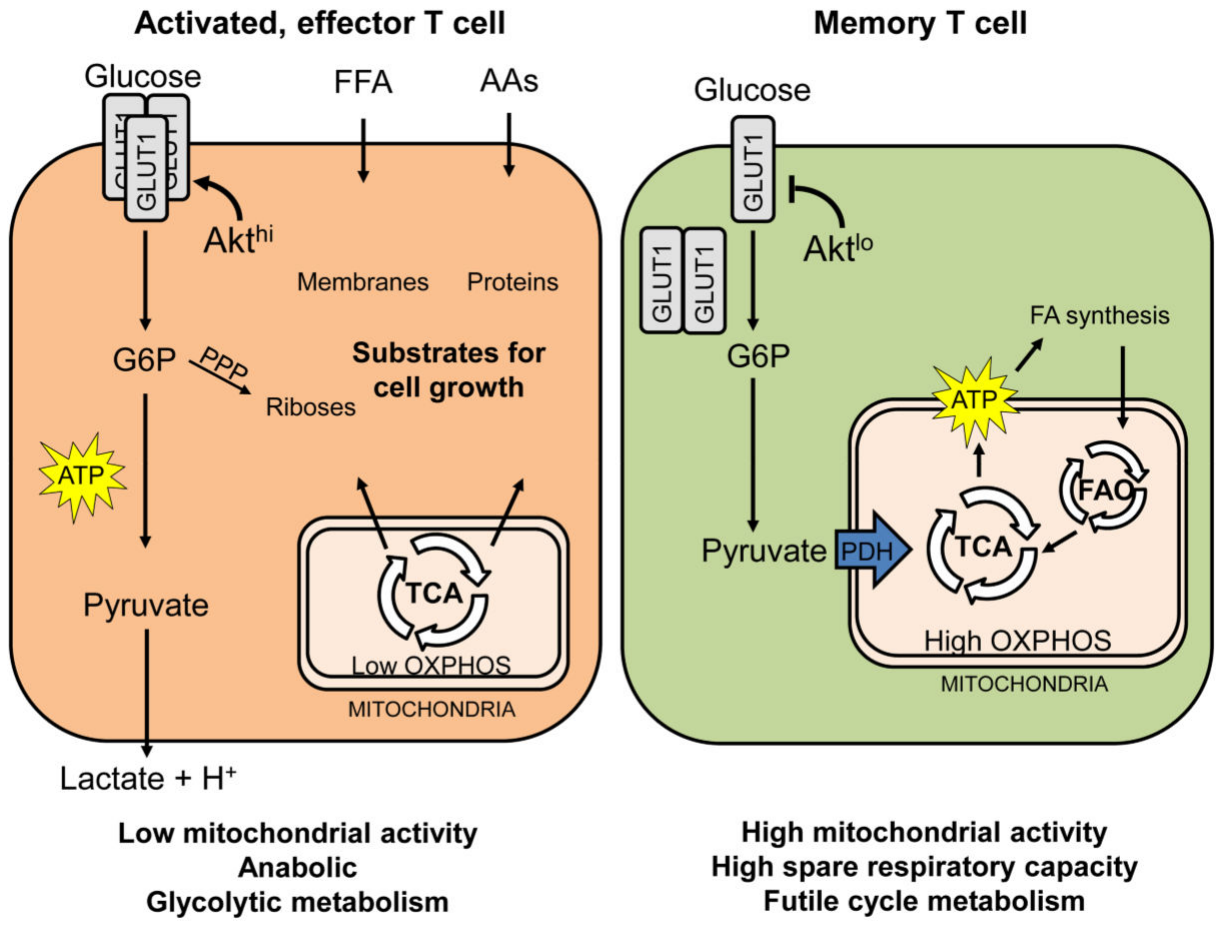
T cells dramatically shift metabolism when in their effector phase During T cell expansion, glucose is preferentially fermented into lactic acid, while other metabolites are used to generate intermediates required for cellular growth and proliferation (left). In periods of quiescence, T cells utilize glucose, amino acids, and fatty acids (intrinsic or extrinsic) in order to generate ATP via oxidative phosphorylation (right). While effector cells activate oxidative phosphorylation during T cell activation, aerobic glycolysis is required for optimal effector cell function and cytokine secretion.

## HIGHLIGHTS

- T cell can utilize many different sources of fuel
- Enforced metabolism or reliance on one pathway can have deleterious effects on T cell function
- Nutrient sensing via mTOR represents a key activation and differentiation signal for T cells
- Other nutrient sensors also play critical roles in T cell fate, in collaboration with or independent of mTOR
